# Supplementary material for: Reactive Case Detection Strategy for Malaria Control and Elimination: A 12 Year Systematic Review and Meta-Analysis from 25 Malaria-Endemic Countries
Source: Trop Med Infect Dis. 2023 Mar 18;8(3):180. doi: 10.3390/tropicalmed8030180 (PMC10058581; doi:10.3390/tropicalmed8030180)
Supplement: Supplementary file 1 [file tropicalmed-08-00180-s001.zip › tropicalmed-2225786-supplementary.pdf]

Table S1: Characteristics of study participants

Study (year), country	No. of Index cases	< 5 years (%)	Male (%)	No. of contacts screened	< 5 years (%)	Male (%)	Average contacts /case	Positive among contacts	Average number of index cases needed to be traced to find one new case	Average number of contacts needed to be screened to find a new case
Dharmawardena *et al.*, 2022 [14], Sri Lanka	70	Not reported	Not reported	77	N/A	72 / 77 (93.5%)	1.1	Microscopy: 3 / 77 (3.9%)	23.3	25.7
Okebe *et al.*, 2021 [15], Gambia	85	Not reported	Not reported	1316	Not reported	Not reported	2	PCR: 67/1316 (5.1%)	1.3	19.6
Roh *et al.*, [16] 2021, Thailand	168	N/A	166/166 (100%). 166 data available for analysis	50	Not reported	Not reported	0.3	Not reported	N/A	N/A
Meredith *et al.*, 2021 [17], Kenya	1,104	Not reported	488/ 1042 (46.8%)	3,353	Not reported	1,380/ 3,183 (43.4%)	3.0	RDT: 365/3167 (11.6%) PCR: 970/3167 (30.6%)	114	3.5

Gunasekera *et al.*, 2021 [18], Sri Lanka	151	Not reported	Not reported	600	Not reported	Not reported	3.9	4	38	150
Stratil, *et al.*, 2021 [19], Cambodia	54	N/A	Not reported	1495	N/A	Not reported	28	1	54	1495
Mkali *et al.*, 2021 [20], Tanzania	21,443	Not reported	Not reported	85,318	Not reported	39616/82021 (48%)	3.9	Not reported	N/A	N/A
Vilakati *et al.*, 2021 [21], Eswatini (Swaziland)	53	Not reported	Not reported	1455	Not reported	Not reported	27.5	RDT: 5/1455 (0.4%) LAMP: 6/1316 (0.5%)	9	243
Morales *et al.*, 2021 [22], Ecuador	2 (RACD 1)<br>2 (RACD 2)<br>1 (RACD 3)	Not reported<br>Not reported<br>Not reported	Not reported<br>Not reported<br>Not reported	167<br>15<br>62	Not reported<br>Not reported<br>Not reported	Not reported<br>Not reported<br>Not reported	83.4<br>7.5<br>62	Microscopy: 3/167 (1.8%)<br>Microscopy: 1/15(6.7%)<br>Microscopy: 2/62(3.2%)	0.6<br>2<br>0.5	55.7<br>15<br>31

Searle, *et al.*, 2021 [23], Zambia	84	N/A	39/84 (46.4%)	1701	N/A	783/1701 (46.0%)	20.3	RDT/PCR: 145/1701 (8.5%)	0.6	11.7
Tessema *et al.*, 2020 [24], Ethiopia	18	Not reported	Not reported	499	Not reported	Not reported	27.7	RDT: 43(8.8%) Pf., 14(2.9%) Pv, 7(1.4%) mixed 111/460 (24.1%) PCR: 47(10.2%) Pf, 55(11.9%) Pv, 9(2.0%)	0.2	4.5
Bridges *et al.*, 2020 [25], Zambia	428	Not reported	Not reported	11,954	Not reported	Not reported	27.9	RDT: 206/11954 (1.7%)	2.1	58
Conner, *et al.*, 2020 [26], Senegal	13	Not reported	Not reported	833	Not reported	Not reported	64.1	RDT: 18/826 (2.2%)	0.7	46.3
Grossenbacher *et al.*,	156	Not reported	Not reported	4590	Not reported	Not reported	29.4	PCR: 78/4590	2	58.8

2020 [27], Tanzania								(1.7%)		
Daniels *et al.*, 2020 [28], Senegal	673	Not reported	Not reported	Not reported	Not reported	Not reported	N/A	RDT: 86 secondary infections	7.8	N/A
Canavati *et al.*, 2020 [29], Vietnam	110	N/A	90/110 (81.8%)	197	N/A	143/197 (72.6%)	1.8	RDT: 0/197 (0.0%)	110	197
Hsiang *et al.*, 2020 [30], Namibia	984	Not reported	Not reported	4701	Not reported	Not reported	4.8	RDT: 114/4701 (2.4%) LAMP: 178/ 4286 (4.2%)	5.5	26.4
Kheang *et al.*, 2020 [31], Cambodia.	408	Not reported	Not reported	1377	Not reported	Not reported	3.4	14/395 (3.5%)	29.1	98.4
Hsiang *et al.*, 2020 [32], Eswatini (Swaziland)	1163	Not reported	Not reported	10 890	Not reported	4399/ 10446 (42.1%)	9.4	RDT: 59/9983 (0.6%) LAMP: 180/10446 (1.7%)	6.5	60.5
Stuck *et al.*, 2020 [33],	418	59/418 (14.1%)	239/418 (57.2%)	17,458	2859 /17458	8087/ 17458	41.8	RDT: 104/12478	2.8	118

Tanzania					(16.4%)	(46.3%)		(0.8%) PCR: 148/6281 (2.4%)		
Bhondoekhan *et al.*, 2020 [34], Zambia	158	Not reported	Not reported	4170	Not reported	Not reported	26.4	RDT/PCR: 153/4170 (3.7%)	1.0	27.3
Bekolo and Williams, 2019 [35], Cameroon	176	132/176 (75.0%)	107/176 (60.8%)	290	Not reported	124 / 249 (49.8%)	1.6	RDT: 249/290 (85.9%)	0.7	1.2
Melese *et al.*, 2019 [36], Ethiopia	20	Not reported	Not reported	270	Not reported	Not reported	13.5	Microscopy: 14/270 (5.2%) PCR: 11/92 (12.0)	1.8	24.5
Aidoo *et al.*, 2018 [37], Kenya	50	Not reported	Not reported	1280	230 (17.9%)	498 (38.9%)	26	PCR: 144/1280 (11%)	0.35	8
Deutsch Feldman *et al.*, 2018 [38], Zambia	145	Not reported	Not reported	3,333	Not reported	Not reported	23	RDT: 33/3016 (1.1%), PCR: 73/3016 (2.4%)	2	46
Kyaw *et al.*, 2018 [39],	312	Not reported	238/312 (76.3)	1185	Not reported	Not reported	4	RDT: 0/1185 (0%)	> 321	> 1284

Myanmar										
Zelman *et al.*, 2018 [40] , Indonesia	36	Not reported	Not reported	1495	Not reported	Not reported	42	PCR: 6/1495 (0.4%)	6	252
Bansil *et al.*, 2018 [41], Ethiopia	220	Not reported	192 (87.3%)	3243	Not reported	Not reported	15	127/3243 (3.9%) RDT: (2.2% P.f., 0.5% Pv, 1.2% mixed)	2	25
Feng *et al.*, 2018 [42] , China	26	Not reported	Not reported	84	Not reported	Not reported	3	4 /84 (4.8%)	6	18
Zemene *et al.*, 2018 [43] Ethiopia	39	Not reported	Not reported	726	144 (19.8%)	322 (44.4%)	19	Microscopy: 29/726 (4.0%) PCR: 54/603 (8.96%)	1	13
Naeem *et al.*, 2018 [44], Pakistan	200	Not reported	Not reported	800	Not reported	Not reported	4.0	Microscopy: 6/800 (0.8%)<br><br>PCR: 40/800 (5.0%)	5.0	20
Zhang *et al.*, 2018 [45], China	863	Not reported	778/863 (90.2%)	41,560	Not reported	Not reported	48.2	10 / 3461 (0.3%)	0.0	4156
Rossi *et al.*, 2018 [46],	194	Not reported	Not reported	785	Not reported	Not reported	4	RDT : 7/ 785 (0.9%)	6	25

Cambodia								PCR : 31/ 785 (3.9%)		
Larsen *et al.*, 2017 [47] , Zambia	854	Not reported	Not reported	14,409	Not reported	Not reported	17	RDT: 1200 / 14409 (8.3%)	0.7	12
Wang *et al.*, 2017 [48], China	260	Not reported	Not reported	3662	Not reported	Not reported	14	RDT: 10/3662 (0.3%)	26	364
Smith *et al.*, 2017 [49], Namibia	146	Not reported	Not reported	3,151	503 (15.9%)	1411 (44.8)	22	RDT : 23/ 3151 (0.7%) LAMP: 47/ 3151 (1.5%)	3	67
Molina Gómez *et al.*, 2017 [50] Colombia	8	Not reported	Not reported	175	Not reported	Not reported	22	Microscopy: 4.6% PCR: 8%	1	12
Tejedor-Garavito *et al.*, 2017 [51], Swaziland	1517	Not reported	Not reported	9859	1813/9754 (18.6%)	6606/9859 (67.0%)	6.5	RDT: 105/9859 (1.1%)	14.4	93.9
Hamze *et al.*, 2016	100	100/100 (100.0%	Not reported	362	Not reported	Not reported	3.6	RDT: 29/362	3.4	12.5

[52], Democratic Republic of Congo		)						(8.0%)		
Hustedt *et al.*, 2016 [53], Cambodia	270	Not reported	Not reported	1898	Not reported	Not reported	7	RDT: 9/1898 (0.5%) PCR: 17/1596 (1.1%)	16	110
Fontoura *et al.*, 2016 [54], Brazil	41	Not reported	Not reported	1,923	Not reported	1,037 (53.9%)	47	Microscopy and PCR: 108 & 293	0.1	6
Chihanga *et al.*, 2016 [55], Botswana	227	Not reported	Not reported	3237	Not reported	Not reported	14.3	37/3237 (1.1%)	6.1	87.5
Donald *et al.*, 2016 [56], Vanuatu	2	Not reported	Not reported	173	Not reported	Not reported	86.5	PCR: 1/173 (0.6%)	2	173
Herdiana *et al.*, 2016 [57], Indonesia	38	Not reported	Not reported	1495	Not reported	653/1495 (43.7%)	39.3	LAMP/ PCR: 6/1495 (0.4%)	6.3	249.2
Searle *et al.*, 2016 [58], Zambia	26	Not reported	Not reported	428	Not reported	Not reported	16	RDT: 50/428 (12%)	1	8
van Eijk *et al.*,	18	0 (0 %)	13 (72.2 %)	868	44 (5.1 %)	342 (39.4	48	Microscopy and PCR:	5	216

2016 [59] , Chennai (India)						%)		4/868 (0.5 %, 3 Pv, 1 Pf)		
van Eijk *et al.*, 2016 [59], Nadiad (India)	20	1 (5.0 %)	14 (70.0 %)	131	2 (1.5 %)	53 (40.5 %)	7	Microscopy and PCR: 0/131 (0.0 %)	>20	>140
Wangdi, *et al.*, 2016 [60], Bhutan	5	Not reported	Not reported	728	Not reported	Not reported	145.6	Not reported	N/A	N/A
Larson *et al.*, 2016 [61], Zambia	Not reported	Not reported	Not reported	167	Not reported	Not reported	N/A	32/167 (19.2%)	N/A	5.2
Pinchoff *et al.*, 2015 [62], Zambia	426	185 (43.4 %)	228 (53.5 %)	1621	Not reported	Not reported	4	RDT: 735/1621 (45.3 %)	1	2
Larsen *et al.*, 2015 [63], Zambia	144	Not reported	Not reported	3955	Not reported	Not reported	27	RDT: 66 / 3955 (1.6 %)	2	58
Littrell *et al.*, 2013 [64] Senegal	110	1 (0.9)	90 (81.8 %)	5520	1021 (18.5 %)	2782 (50.4 %)	50	RDT: 23/5520 (0.4 %)	5	240
Sturrock *et al.*,	247	Not reported	151 (61.1)	3671	163 (4.4 %)	2168 (59.1 %)	15	RDT: 74/3671	3	50

2013 [8], Swaziland								(2.0 %)		
Rogawski *et al.*, 2012 [65] Thailand	1	Not reported	Not reported	126	Not reported	Not reported	126	Microscopy: 1/126 (0.8 %, Pf). PCR: 2/126 (1.6 %, 1 Pf, 1 Pv)	1	63
Stresman *et al.*, 2010 [66] Zambia	23	Not reported	Not reported	186	46 (24.7 %)	86 (46.2 %)	8	RDT: 5/185 (2.7 %). PCR: 7/186 (3.4 %)	5	37

Pv: *Plasmodium vivax*; Pf: *Plasmodium falciparum*; RDT: Rapid Diagnostic Test; PCR: Polymerase Chain Reaction; LAMP: Loop - Mediated Isothermal Amplification
